# Characterization of substrates and inhibitors of the human heterodimeric transporter 4F2hc-LAT1 using purified protein and the scintillation proximity radioligand binding assay

**DOI:** 10.3389/fphys.2023.1148055

**Published:** 2023-02-21

**Authors:** Satish Kantipudi, Daniel Harder, Dimitrios Fotiadis

**Affiliations:** Institute of Biochemistry and Molecular Medicine, University of Bern, Bern, Switzerland

**Keywords:** amino acid transporter, JPH203, LAT1, scintillation proximity assay, SLC7, inhibitor, membrane protein, *Pichia pastoris*

## Abstract

Amino acids have diverse and essential roles in many cellular functions such as in protein synthesis, metabolism and as precursors of different hormones. Translocation of amino acids and derivatives thereof across biological membranes is mediated by amino acid transporters. 4F2hc-LAT1 is a heterodimeric amino acid transporter that is composed of two subunits belonging to the SLC3 (4F2hc) and SLC7 (LAT1) solute carrier families. The ancillary protein 4F2hc is responsible for the correct trafficking and regulation of the transporter LAT1. Preclinical studies have identified 4F2hc-LAT1 as a valid anticancer target due to its importance in tumor progression. The scintillation proximity assay (SPA) is a valuable radioligand binding assay that allows the identification and characterization of ligands of membrane proteins. Here, we present a SPA ligand binding study using purified recombinant human 4F2hc-LAT1 protein and the radioligand [^3^H]L-leucine as tracer. Binding affinities of different 4F2hc-LAT1 substrates and inhibitors determined by SPA are comparable with previously reported *K*_m_ and *IC*_50_ values from 4F2hc-LAT1 cell-based uptake assays. In summary, the SPA is a valuable method for the identification and characterization of ligands of membrane transporters including inhibitors. In contrast to cell-based assays, where the potential interference with other proteins such as endogenous transporters persists, the SPA uses purified protein making target engagement and characterization of ligands highly reliable.

## 1 Introduction

Amino acids are the building blocks of proteins and play critical roles in the human body, e.g., as nutrients, metabolites, precursors of hormones and signaling molecules (Wu, 2009). Specific and controlled transport of amino acids through biological membranes is of fundamental physiological importance and mediated by amino acid transporters (AATs) that are embedded in lipid bilayers (Christensen, 1990; McGivan and Pastor-Anglada, 1994). Consequently, the absence, overexpression or dysfunction of AATs can lead to human diseases (Bröer and Palacin, 2011). Currently, eleven solute carrier (SLC) families containing AATs were reported (Kandasamy et al., 2018). Based on their substrate specificity and mechanism of transport, AATs are classified into different systems (Christensen, 1990; McGivan and Pastor-Anglada, 1994).

Heterodimeric amino acid transporters (HATs) are structurally unique among amino acid transporters from the SLC superfamily as they are composed of a heavy and a light subunit, which are connected *via* a disulfide bridge (Nakamura et al., 1999; Chillaron et al., 2001; Verrey et al., 2004; Fotiadis et al., 2013). Mutations in HATs are associated with inherited metabolic diseases such as aminoacidurias (e.g., cystinuria and lysinuric protein intolerance) (Chillaron et al., 2001; Verrey et al., 2004; Fotiadis et al., 2013), viral infections (Kaleeba and Berger, 2006; Rabinowitz et al., 2021) and tumor growth (Fotiadis et al., 2013; Häfliger and Charles, 2019; Lu, 2019; Saito and Soga, 2021; Kanai, 2022). Heavy subunits are type II membrane N-glycoproteins and belong to the SLC3 family (Palacin and Kanai, 2004; Verrey et al., 2004; Fotiadis et al., 2013). On the other hand, the light and catalytic subunits of HATs are L-type amino acid transporters (LATs) from the SLC7 family (Reig et al., 2002; Rosell et al., 2014; Napolitano et al., 2015). The two heavy subunits 4F2hc (SLC3A2, CD98) and rBAT (SLC3A1) ensure the correct trafficking of LATs to the plasma membrane in mammalian cells (Nakamura et al., 1999; Chillaron et al., 2001; Verrey et al., 2004; Fotiadis et al., 2013). Recently, an additional and novel function of 4F2hc in modulating the substrate affinity and specificity of specific HATs was unveiled (Kantipudi et al., 2020).

The light subunit LAT1 (SLC7A5) (Scalise et al., 2018) is expressed mainly in organs such as the brain, placenta, spleen and testis (Nakamura et al., 1999; Prasad et al., 1999). Furthermore, LAT1 is overexpressed in numerous cancer types, e.g., brain (Nawashiro et al., 2006), breast (Kurozumi et al., 2022), gastric (Ichinoe et al., 2011), lung (Kaira et al., 2008), pancreatic (Kaira et al., 2012), prostate (Sakata et al., 2009), renal cell (Betsunoh et al., 2013) and urologic cancer (Nakanishi et al., 2007) (also see (Wang and Holst, 2015) and (Häfliger and Charles, 2019) for LAT1 expression in other tumors), and is used as a pathological factor for an unfavorable prognosis in patients. In such cancer cells, the nutritional uptake of neutral and essential neutral amino acids, and co-regulation of the mammalian target of rapamycin (mTOR) signaling pathway is mediated by the LAT1 transporter (Nicklin et al., 2009). Because of its tissue distribution and expression levels, the LAT1 transporter has become an interesting vehicle for drug delivery into the brain and cancer cells (Wang and Holst, 2015; Singh et al., 2018; Häfliger and Charles, 2019; Scalise et al., 2021; Kanai, 2022).

LAT1 is part of the amino acid transport system L (Verrey, 2003). It transports large neutral amino acids with branched or aromatic side chains (Kanai et al., 1998; Mastroberardino et al., 1998; Yanagida et al., 2001; Meier et al., 2002) and has relatively high affinities for L-leucine and L-histidine (Mastroberardino et al., 1998; Yanagida et al., 2001; Napolitano et al., 2015; Kantipudi et al., 2020; Kantipudi and Fotiadis, 2021). It is a Na^+^-independent obligatory exchanger that transfers one substrate molecule out of the cell in exchange for another into the cell with 1:1 stoichiometry (Verrey et al., 2004; Fotiadis et al., 2013). Substrates of LAT1 are, besides large neutral L-amino acids, the Parkinson’s and anticonvulsant drugs L-DOPA (Kageyama et al., 2000) and gabapentin (Uchino et al., 2002), as well as the thyroid hormones triiodothyronine (T3) and thyroxine (T4) (Friesema et al., 2001; Zevenbergen et al., 2015). The broad system L inhibitor 2-aminobicyclo-(2,2,1)-heptane-2-carboxylic acid (BCH) also inhibits LAT1 (Kanai et al., 1998). In contrast, the compound JPH203 (KYT-0353), a tyrosine-analog, specifically and strongly inhibits the LAT1 transporter and has shown important inhibitory effects on the growth of different cancer cells (Oda et al., 2010; Yun et al., 2014). Moreover, JPH203 treatment arrested *in vivo* the tumor growth in a fully immunocompetent mouse model of thyroid cancer (Häfliger et al., 2018).

The methylotrophic yeast *Pichia pastoris* is a well-established cellular factory for the production of recombinant mammalian membrane proteins (Byrne, 2015; Looser et al., 2015; Pochini and Galluccio, 2022). Over the years, different human LATs and HATs were successfully overexpressed in *P. pastoris* for functional and structural studies (Costa et al., 2013; Meury et al., 2014; Rosell et al., 2014; Jeckelmann and Fotiadis, 2019; 2020; Kantipudi et al., 2020; Kantipudi et al., 2021; Kantipudi and Fotiadis, 2021; Jeckelmann et al., 2022). For example, cell-based transport assays using *P. pastoris* overexpressing 4F2hc-LAT1 were established and used for the characterization of the specificity for substrates and inhibitors, and their kinetic parameters (Kantipudi et al., 2020; Kantipudi and Fotiadis, 2021). Recently, a protocol for the production in *P. pastoris* and purification of human 4F2hc-LAT1 was reported that allows the isolation of milligram amounts of pure, correctly assembled, stable and properly folded heterodimer (Kantipudi et al., 2021).

For discovering and characterizing membrane transporter-specific ligands, direct, rapid and robust binding assays are of great advantage. Such a binding assay represents the scintillation proximity radioligand binding assay (SPA) (Quick and Javitch, 2007; Harder and Fotiadis, 2012). In the SPA, purified, detergent-solubilized target transporter is bound to scintillation beads and the radiolabeled substrate is added. Substrate binding to the transporter will induce photon emission from the SPA beads, because of the close proximity of the protein-bound radioligand to the scintillant. The luminescence signal is measured and reflects substrate binding. Such an assay has the advantage of being free of endogenous transporters and other proteins, e.g., as present in cell-based assays, thus delivering a clean target protein-ligand interaction-specific information.

Here, we present an extensive ligand-binding study for human 4F2hc-LAT1. The recombinant HAT was overexpressed in *P. pastoris*, purified and characterized using the SPA. Binding specificities and affinities of selected substrates and inhibitors were successfully determined for human 4F2hc-LAT1. The obtained results were compared and validated with previously published data, e.g., from cell-based transport assays, and are discussed, establishing the SPA as an excellent, robust ligand-binding assay for HATs.

## 2 Materials and methods

### 2.1 Cloning and selection of 4F2hc-LAT1

Cloning of the human HAT 4F2hc-LAT1 into the pPICZB vector (Thermo Fisher Scientific, Waltham, MA, USA) and transformation into electro-competent *P. pastoris* strain KM71H cells by electroporation was described previously (Kantipudi et al., 2020). This construct contains recombinant human 4F2hc-LAT1 bearing N-terminal His- (4F2hc) and Strep- (LAT1) tags. The screening and selection protocol of 4F2hc-LAT1 protein expression clones was detailed in (Kantipudi et al., 2021). Large-scale overexpression in *P. pastoris*, membrane isolation and protein purification of human 4F2hc-LAT1 using the detergent glyco-diosgenin (GDN, Anatrace) were performed as described in detail in (Kantipudi et al., 2021).

### 2.2 Scintillation proximity radioligand binding assay experiments

**FIGURE 1:**
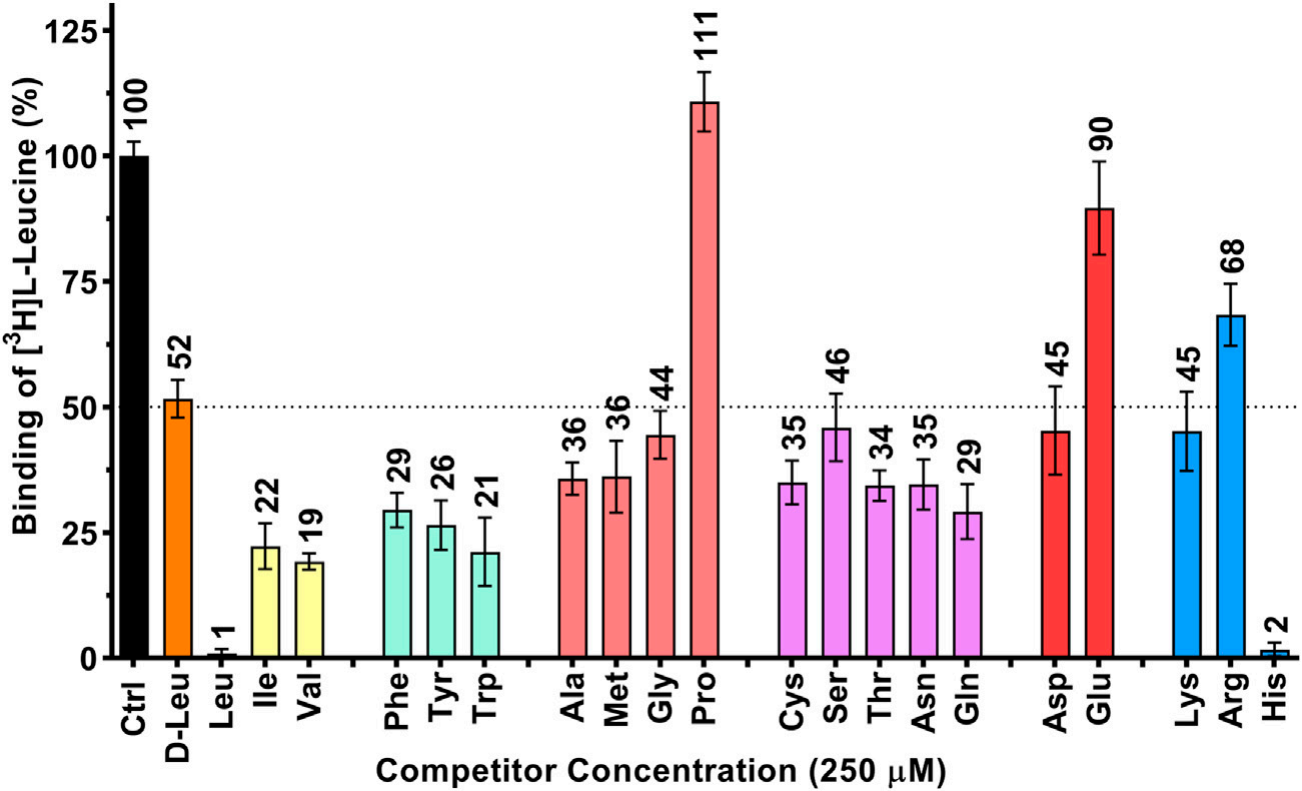
Determination of the human 4F2hc-LAT1 binding specificity for selected amino acids by SPA. All twenty proteogenic L-amino acids and D-leucine were used as competitors. A competitor concentration of 250 µM was used, which corresponds to ten times the K_m_ of 4F2hc-LAT1 for L-leucine (Kantipudi et al., 2020). Residual binding of the radioligand [^3^H]L-leucine in the presence of competitors was normalized with respect to control samples without competitors (Ctrl). Means with SD from normalized data of three independent experiments (each at least in triplicate) are displayed. The numbers above the bars represent the mean values in %.

**FIGURE 2:**
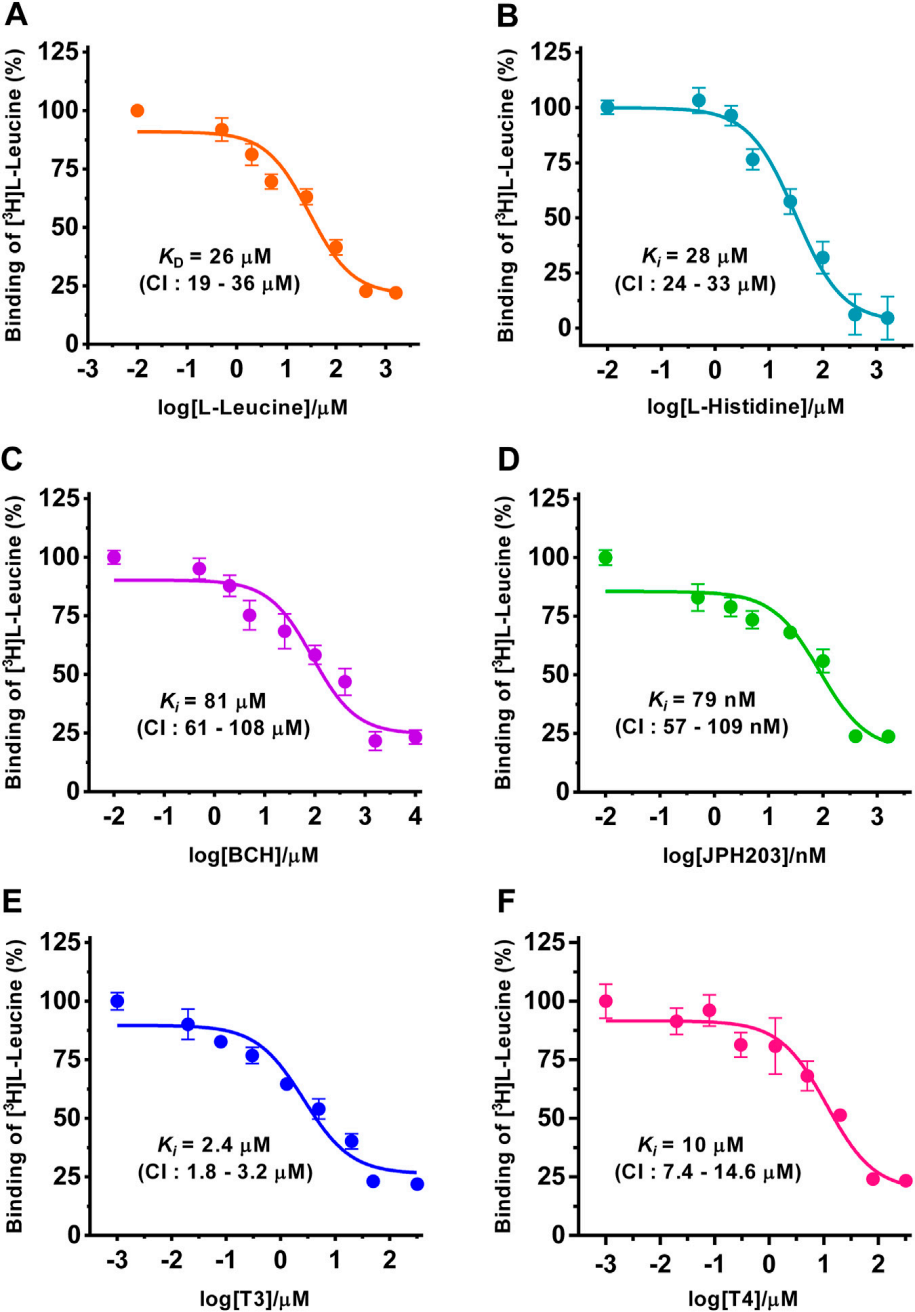
Determination of dissociation (*K*_D_) and inhibition constants (*K*_i_) of selected substrates and inhibitors for human 4F2hc-LAT1 by SPA. Competitive binding curves for determination of *K*_D_ [L-leucine **(A)**, orange], and *K*_i_ values [L-histidine **(B)**, light blue; BCH **(C)**, violet; JPH203 **(D)**, green; T3 **(E)**, blue and T4 **(F)**, dark pink]. Determined *K*_D_ and *K*_i_ values, and 95% confidence intervals (CIs) are indicated. Mean ± standard deviation of normalized data from three independent experiments, each at least in triplicate, are shown. If not visible, error bars are smaller than symbols.

**FIGURE 3:**
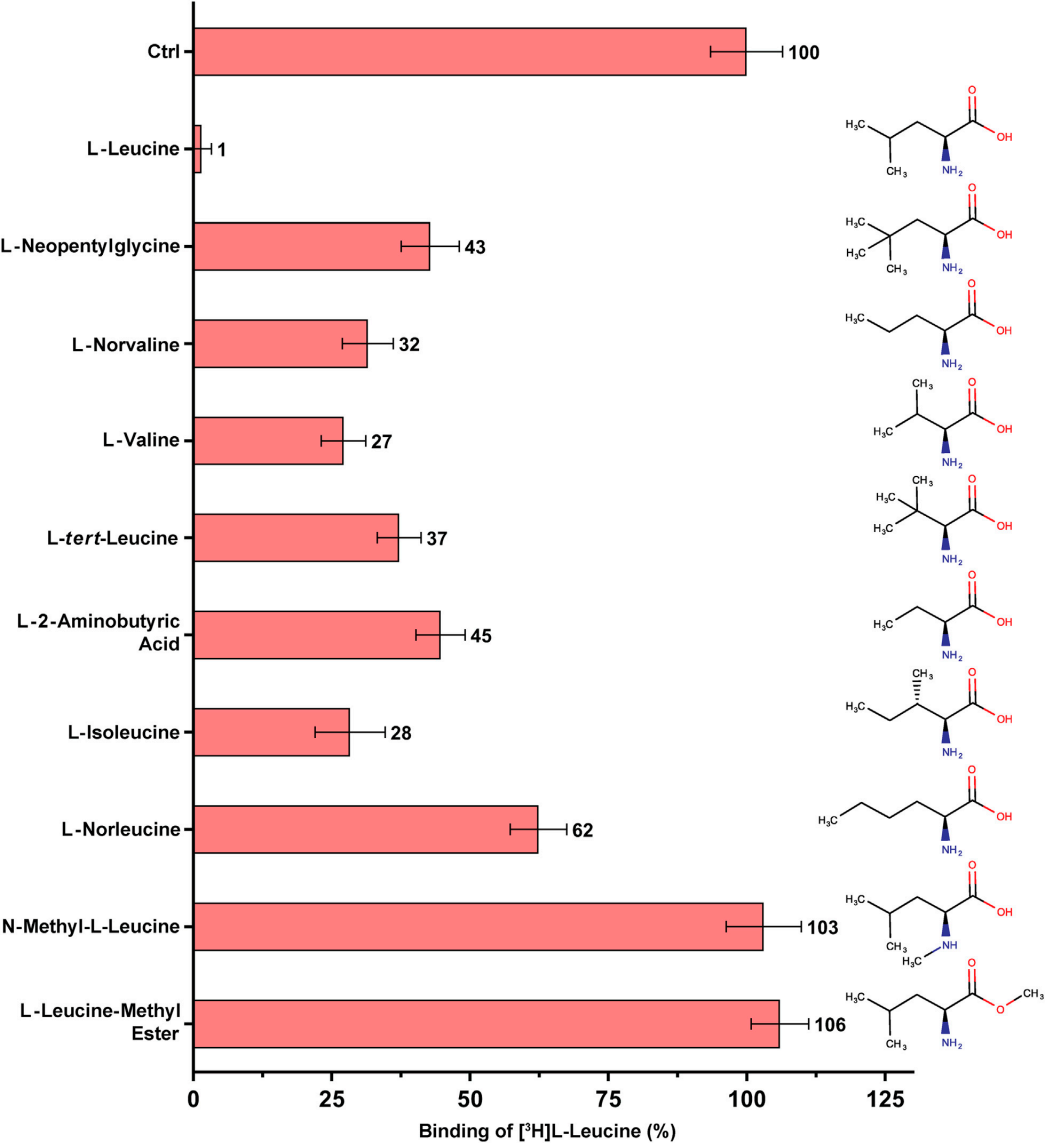
SPA binding competition experiments for l-leucine and selected structural analogues, e.g., with varying aliphatic chain lengths and number of methyl groups. Purified 4F2hc-LAT1 and [^3^H]L-leucine were used for SPA as described in Materials and Methods. The competitor concentration was 250 µM. Residual binding in the presence of competitors was normalized with respect to control samples without competitors (Ctrl). Means with SD from normalized data of three independent experiments (each at least in triplicate) are shown. The numbers next to the bars represent the mean values in %.

**FIGURE 4:**
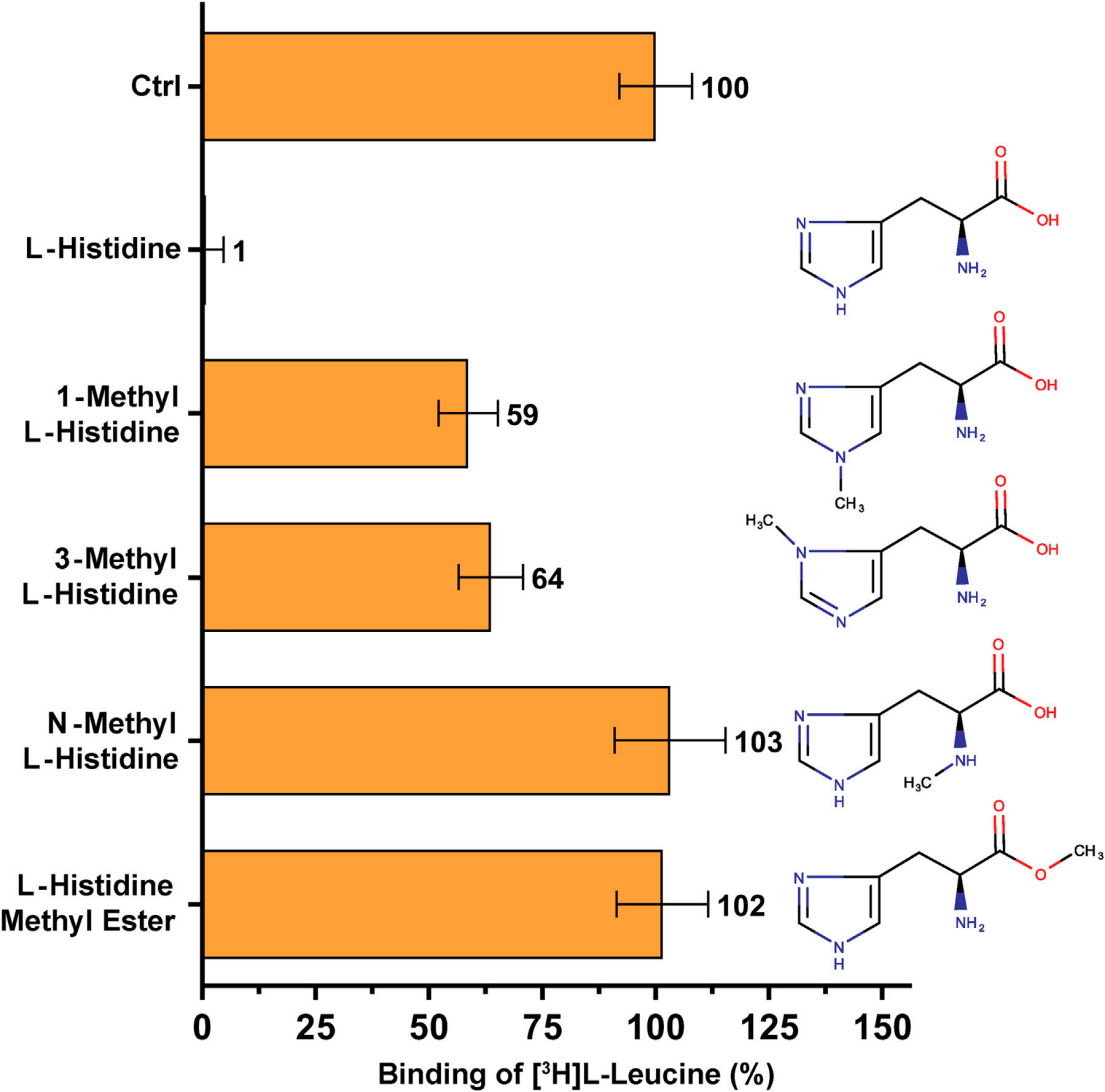
SPA binding competition experiments for l-histidine and selected structural analogues, i.e., molecules with a methyl group at different positions in the molecule. Purified 4F2hc-LAT1 and [^3^H]L-leucine were used for SPA as described in Materials and Methods. The competitor concentration was 250 µM. Residual binding in the presence of competitors was normalized with respect to control samples without competitors (Ctrl). Means with SD from normalized data of three independent experiments (each at least in triplicate) are shown. The numbers next to the bars represent the mean values in %.

Purified, GDN-solubilized human 4F2hc-LAT1 was attached *via* the His-tag to polyvinyltolune (PVT) copper His-tag SPA beads (Perkin Elmer) and the protein-bound [^3^H]L-leucine quantified using a scintillation counter (a detailed description of SPA with 4F2hc-LAT1 is given in the methodological publication by (Kantipudi et al., 2021)). Briefly, experiments were conducted in 96-well plates and per well a reaction volume of 50 µL containing 250 µg PVT-SPA-beads, 1.75 µg 4F2hc-LAT1 protein, 2.5 μM L-leucine spiked with 0.5 µCi [^3^H]L-leucine (ARC/Anawa, 100 Ci/mmol, 1 mCi/ml) plus a condition-specific substance. All components were dissolved in SPA-Buffer (100 mM BTP pH 8, 150 mM NaCl, 10% (v/v) glycerol, 0.5% (w/v) GDN). For some condition-specific substances (i.e., the thyroid hormones), 20 min sonication in a water bath at 4°C was necessary to dissolve them at 10× concentration in SPA-Buffer. Per reaction, 5 µL of a 10× stock solution of the condition-specific substance was placed in a well and diluted with 45 µL of a master mix containing the remaining components. For determining the substrate specificities (Figure 1), the twenty proteogenic L-amino acids and D-leucine were used as competitors at a final concentration of 250 µM. For determining the *K*_D_ of L-leucine (Figure 2A) and *K*_i_ of L-histidine (Figure 2B), homologous (L-leucine) and heterologous (L-histidine) substrate competition was applied at concentrations ranging from 0.01 to 1600 µM. For *K*_i_ determination of the system L inhibitor BCH, concentrations ranging from 0.01 to 10,000 µM BCH as competitor were used (Figure 2C). The binding kinetics of hydrophobic compounds such as JPH203 [(S)-2-amino-3-(4-((5-amino-2-phenylbenzo [d] oxazol-7-yl)-methoxy)-3,5-dichlorophenyl)-propanoic acid] also known as KYT-0353 (MedChemExpress, Monmouth Junction, NJ, United States), and the thyroid hormones T3 (triiodothyronine) and T4 (thyroxine) (Sigma, St. Louis, MO, United States) were also determined by SPA. For *K*_i_ values of hydrophobic compounds, final ligand concentrations of 0.01–1600 nM for JPH203 (Figure 2D) and 0.001-320 µM for T3 and T4 (Figures 2E, F) were used. The binding specificities of structural analogues of L-leucine (Figure 3) and L-histidine (Figure 4) were tested at a final competitor concentration of 250 µM. The amino acid analogues of L-leucine and L-histidine displayed in Figures 3, 4 were from Sigma (Sigma St. Louis, MO, United States). To avoid minor unspecific binding of radioactive L-leucine to the beads, the master mix was created in a specific procedure: SPA-beads were first mixed with the unlabeled L-leucine and shaken for 2 h at 4°C before adding the remaining components (protein last). For background subtraction in the bar graph experiments, a no-protein control was included using protein-free master mix (i.e., containing buffer instead of protein solution). The plate was shortly mixed by shaking and incubated at 4°C for ∼18 h before the signals were counted using a microplate scintillation counter (Trilux Microbeta, Perkin Elmer). [^3^H]L-leucine protein (full signal) and no-protein (background) SPA samples had typically ∼3600 and ∼800 CPM, respectively. Thus, ∼20% of the full signal was background. Three experiments with protein from at least two different purifications were performed, each at least in triplicates.

### 2.3 Data analysis, curve fitting and statistics

Data were analyzed with Prism 6 (GraphPad Software). In each of these experiments, the net binding signals were averaged and the *K*_D_ value of homologous (L-leucine) and *K*_i_ values of heterologous (L-histidine, BCH, JPH203, T3 and T4) L-leucine binding competition experiments were determined by fitting the respective sigmoidal model curve to these data. Each experiment was done with sample number of at least three (triplicate). Data of three independent experiments were merged by adding the normalized data to a common data set, which was used for curve fitting. For data analysis of the bar graphs, the signal of the no-protein control was subtracted from the transporter signal to obtain the net binding signal.

## 3 Results and discussion

An extensive ligand binding study was performed to evaluate the potential of the SPA on HATs using human 4F2hc-LAT1 as a paradigm. To this aim, recombinant heterodimer was overexpressed in the methylotrophic yeast *P. pastoris* and purified. Previous work from our laboratory demonstrated that recombinant human 4F2hc-LAT1 expressed and isolated from *P. pastoris* is correctly assembled, fully functional (Kantipudi et al., 2020; Kantipudi et al., 2021; Kantipudi and Fotiadis, 2021), pure and stable in detergent (Kantipudi et al., 2021). As radioligand for SPA experiments, [^3^H]L-leucine was chosen based on our recent results from uptake studies using *P. pastoris* cells expressing human 4F2hc-LAT1 (Kantipudi et al., 2020; Kantipudi and Fotiadis, 2021). These previous functional studies identified L-leucine as a high affinity substrate.

In a first set of experiments, the specificity of 4F2hc-LAT1 for the twenty proteinogenic amino acids and D-leucine was explored using purified protein and the SPA. Results from [^3^H]L-leucine binding inhibition to 4F2hc-LAT1 on SPA beads (Figure 1) indicated a pattern comparable to our previously published [^3^H]L-leucine uptake inhibition study (Kantipudi et al., 2020), validating the here presented SPA approach. For example, L-leucine and L-histidine inhibited strongly the binding of [^3^H]L-leucine to 4F2hc-LAT1. The aliphatic amino acids L-isoleucine and L-valine as well as the aromatic ones, i.e., L-phenylalanine, L-tyrosine and L-tryptophan, indicated with residual radioligand binding of 19-29% comparable and significant inhibitions of [^3^H]L-leucine binding. Situated at the upper end of this range, L-glutamine indicated with 29% residual [^3^H]L-leucine binding an inhibition comparable to L-phenylalanine. Considering that 4F2hc-LAT1 is involved in the exchange of L-glutamine from inside with L-leucine from outside for intracellular L-leucine mediated mTOR activation (Nicklin et al., 2009), this significant observation makes sense. In stark contrast and again similar to uptake inhibition, the imino acid L-proline did not show any radioligand binding inhibition (Kantipudi et al., 2020; Kantipudi and Fotiadis, 2021). The other proteinogenic amino acids reduced the residual SPA signal to values between 34 and 90% indicating relatively low substrate specificities. Finally, and similar to results from previous uptake experiments (Kantipudi et al., 2020), the D-form of leucine showed about 50% inhibition indicating stereoselective binding of leucine to human 4F2hc-LAT1.

Next, and also for direct comparison with our previously published [^3^H]L-leucine uptake inhibition data using yeast cells expressing human 4F2hc-LAT1 (Kantipudi et al., 2020; Kantipudi and Fotiadis, 2021), we determined the *K*_D_ and *K*_i_ values of selected substrates and inhibitors, i.e., of L-leucine, L-histidine, BCH, JPH203, triiodothyronine (T3) and thyroxine (T4) using the SPA (Figure 2). For the two best amino acid substrates, we obtained *K*_D_ and *K*_i_ values of 26 µM (L-leucine) and 28 µM (L-histidine), respectively. These numbers are in good agreement with the previously obtained *K*_m_ and *IC*_50_ values of 25 µM (Kantipudi et al., 2020) and 22 µM (L-leucine) (Kantipudi and Fotiadis, 2021), and an *IC*_50_ value of 23 µM (L-histidine) (Kantipudi et al., 2020) using our *P. pastoris* cell uptake assay. It should be noted that the *K*_i_ value of L-histidine (Figure 2B) is also in good agreement with the external *K*_m_ value of L-histidine determined using proteoliposomes reconstituted with recombinant human LAT1 (Napolitano et al., 2015). Considering that SLC7 family transporters have external and internal *K*_m_s for their substrates (Meier et al., 2002; Napolitano et al., 2015; Bartoccioni et al., 2019; Errasti-Murugarren et al., 2019), e.g., the external and internal *K*_m_ values of 24.6 µM and 2.8 mM for L-histidine as reported in the previously mentioned study using proteoliposomes (Napolitano et al., 2015), our ligand-binding parameters suggest that the external side of 4F2hc-LAT1 is accessible to the here studied ligands when the protein is solubilized and purified with the detergent GDN. For the thyroid hormones T3 and T4, *K*_i_ values of 2.4 µM and 10 µM were obtained by SPA. These binding constants were comparable or identical to the previously obtained *IC*_50_ values of 1.3 µM (T3) and 10 µM (T4) from [^3^H]L-leucine uptake inhibition experiments (Kantipudi and Fotiadis, 2021). From the inhibitors, the well-known system L and the human 4F2hc-LAT1 specific inhibitors BCH and JPH203, delivered *K*_i_ values of 81 µM and 79 nM, respectively. Again, the obtained binding constants were comparable with the *IC*_50_ values of 72 µM (BCH) and 197 nM (JPH203) from [^3^H]L-leucine uptake inhibition experiments (Kantipudi et al., 2020; Kantipudi and Fotiadis, 2021).

In summary, *K*_D_ and *K*_i_ values from the selected substrates and inhibitors L-leucine, L-histidine, BCH, JPH203, T3 and T4 were in good agreement with the kinetic parameters from the same molecules using a radioligand cell-based 4F2hc-LAT1 uptake assay (Kantipudi et al., 2020; Kantipudi and Fotiadis, 2021). Thus, the here presented comparative study validated the SPA for new 4F2hc-LAT1 ligand binding experiments.

The amino acid substrates L-leucine and L-histidine showed the highest affinities towards human 4F2hc-LAT1 (Figures 2A,B). Therefore, we decided to explore the possibility of identifying new, non-proteinogenic amino acids and derivatives of L-leucine and L-histidine with higher affinities towards human 4F2hc-LAT1.

To this end, SPA competition binding experiments with [^3^H]L-leucine versus L-leucine and selected non-proteinogenic amino acid were performed (Figure 3). The selected non-proteinogenic amino acid derivatives of L-leucine differ in the number of methyl groups at the γ-carbon atom or in the aliphatic chain lengths with methyl groups at different positions. L-leucine (with two methyl groups at γ-position) inhibited strongly the binding of [^3^H]L-leucine with only ∼1% residual radioligand binding (RRB) (Figure 3). The non-proteinogenic amino acid L-neopentyl glycine (with three γ-methyl groups) and L-norvaline (with one γ-methyl group) showed a weaker inhibition with 32–43% RRB (Figure 3). This indicates that with addition or deletion of one methyl group at the γ-position of L-leucine the binding affinity towards 4F2hc-LAT1 decreases drastically. L-valine, with two methyl groups at the β-position instead of γ-position, showed with 27% RRB less competition than L-leucine (Figure 3). Altering the methyl groups on β-position as in the non-proteinogenic amino acids L-*tert*-leucine (three β-methyl groups) or L-2-aminobutyric acid (one β-methyl group) showed a further decrease of competition potential with 37–45% RRB (Figure 3). The amino acid L-isoleucine, which is an isostereomer of L-leucine is with 28% RRB in a similar range as L-valine (Figure 3). Therefore, the position of the methyl group in the side chains of L-isoleucine and L-leucine, i.e., β-versus γ-position, has a significant effect on the binding to 4F2hc-LAT1. Further increasing the aliphatic chain length by one methyl group with the non-proteinogenic amino acid L-norleucine decreased the competition potential to 62% RRB (Figure 3).

A second, comparable competition SPA experiment was performed to investigate the high-affinity substrate L-histidine and selected non-proteinogenic amino acid derivatives (Figure 4). The amino acid L-histidine inhibits [^3^H]L-leucine binding to only ∼1% RRB (Figure 4). Addition of a methyl group at nitrogen atom either at position 1 (1-methyl-L-histidine) or at position 3 (3-methyl-L-histidine) on the imidazole ring of L-histidine decreases radioligand binding to 4F2hc-LAT1 drastically, resulting in 59–64% RRB binding (Figure 4).

Binding studies with methylated forms of L-leucine and L-histidine amino acids such as N-methyl-L-leucine, L-leucine-methyl ester, N-methyl-L-histidine and L-histidine-methyl ester clearly show that there is no significant binding to 4F2hc-LAT1 (Figures 3, 4). These results indicate that a free amino and carboxy group at the α-carbon of L-leucine and L-histidine are essential for binding to human 4F2hc-LAT1.

## 4 Conclusion

The scintillation proximity radioligand binding assay is a powerful method to determine the specificity and affinity of ligands towards a specific target protein. Here, we successfully determined binding specificities and affinities of selected substrates and inhibitors for the human heterodimeric 4F2hc-LAT1 transporter by applying the SPA. As radioligand for the assay, the high affinity amino acid [^3^H]L-leucine was used together with purified 4F2hc-LAT1 protein that was expressed in the methylotrophic yeast *P. pastoris*. The obtained ligand inhibition pattern (Figure 1), and *K*_D_ and *K*_i_ values (Figure 2) were compared with previously determined data and *IC*_50_ values of the same substrates and inhibitors using an uptake assay with human 4F2hc-LAT1 overexpressing *P. pastoris* cells (Kantipudi et al., 2020; Kantipudi and Fotiadis, 2021). Protein-ligand parameters from both assays were similar, validating the SPA for future applications with human 4F2hc-LAT1 and enabling the focus on ligand binding. Beyond validation and using this SPA experimental set-up, derivatives of the high-affinity 4F2hc-LAT1 substrates l-leucine and l-histidine were tested (Figures 3, 4). Thus, the SPA also represents an excellent ligand-binding assay to screen for potential new substrates and inhibitors of 4F2hc-LAT1 and possibly other HATs.

## Data Availability

The raw data supporting the conclusion of this article will be made available by the authors, without undue reservation.
